# What drives outcomes in infants of mothers with congenital heart disease? A mediation analysis

**DOI:** 10.1038/s41372-023-01796-0

**Published:** 2023-10-19

**Authors:** Brian T. Young, Rebecca J. Baer, Christina D. Chambers, Shabnam Peyvandi, Laura L. Jelliffe-Pawlowski, Martina A. Steurer

**Affiliations:** 1 Department of Pediatrics, University of California San Francisco, San Francisco, CA US; 2 Division of Pediatric Critical Care, Department of Pediatrics, Kaiser Permanente Northern California, Oakland, CA US; 3 Department of Epidemiology and Biostatistics, University of California San Francisco, San Francisco, CA US; 4 Department of Pediatrics, University of California San Diego, La Jolla, CA US

**Keywords:** Paediatrics, Congenital heart defects, Outcomes research, Risk factors, Epidemiology

## Abstract

**Objective:**

Infants of mothers with adult congenital heart disease (ACHD) are at increased risk for adverse pregnancy and neonatal outcomes. We aim to identify mediators in the relationship between ACHD and pregnancy and infant outcomes.

**Study design:**

Case-control study using linked maternal and infant hospital records. Structural equation modeling was performed to assess for potential mediators of pregnancy and infant outcomes.

**Result:**

We showed an increased risk of multiple adverse infant and pregnancy outcomes among infants born to mothers with ACHD. Maternal placental syndrome and congestive heart failure were mediators of prematurity. Prematurity and critical congenital heart disease in the infant were mediators of infant outcomes. However, the direct effect of ACHD on outcomes beyond that explained by these mediators remained significant.

**Conclusion:**

While significant mediators of infant and pregnancy outcomes were identified, there was a large direct effect of maternal ACHD. Further studies should aim to identify more factors that explain these infants’ vulnerability.

## Introduction

Congenital heart disease (CHD) is the most common major congenital malformation, occurring in approximately 9 in every 1000 live births [1]. Advances in the care of these infants have led to increased survival to adulthood; greater than 90% these children are reaching childbearing age, and there are now more adults with CHD than children [1–5].

Due to the hemodynamic changes that occur during pregnancy, women with adult congenital heart disease (ACHD) are known to be at increased risk for adverse cardiovascular and obstetric events [6–10]. Previous research has largely focused on the outcome of mothers with ACHD. A better understanding of infant outcomes is an important part of counseling the increasing numbers of pregnant mothers with ACHD. It has been shown that infants of mothers with ACHD are at greater risk for adverse outcomes. This risk is even higher in mothers with complex congenital heart disease and related complications such as maternal cyanosis and pulmonary hypertension [8–18].

The adverse outcomes can be divided into adverse pregnancy outcomes affecting the infant such as prematurity or the presence of congenital heart disease, and adverse infant outcomes such as length of hospital stay or infant mortality. Different factors affect these two outcome groups: pregnancy conditions might affect prematurity, whereas prematurity in itself will affect future outcomes such as infant mortality.

Thus, the goal of this study is to investigate the complex interplay between potential mediators, pregnancy and infant outcomes.

One proposed mechanism to explain adverse pregnancy outcomes is that mothers with ACHD have abnormal uteroplacental circulation and placental oxygen delivery due to impaired cardiac output, leading to an abnormal placental environment [17, 19]. Several maternal comorbidities are known to adversely affect the placental environment and subsequently fetal health (i.e. maternal placental syndrome [MPS], defined as maternal preeclampsia, gestational hypertension, or placental abruption) [20–22], maternal metabolic syndrome (MMS) [23–26], and maternal infection [27, 28]. Adverse infant outcomes might be mediated by prematurity, CHD of the offspring or an increased rate of malformations or genetic syndromes. Existing literature has focused on outcomes in the neonatal period, and less on outcomes beyond the neonatal period [14, 15, 29].

We utilized mediation analysis and structural equation modeling to elucidate the interplay between ACHD, maternal comorbidities, pregnancy, neonatal and infant outcomes. First, we hypothesized that adverse pregnancy outcomes such as prematurity and having an offspring with critical congenital heart disease (CCHD) are mediated by maternal conditions such as MPS, MMS, congestive heart failure (CHF), and pregnancy infection. Second, we hypothesized that increased infant mortality in the first year of life and morbidity are mediated by prematurity and congenital anomalies.

## Methods

**Fig. 1:**
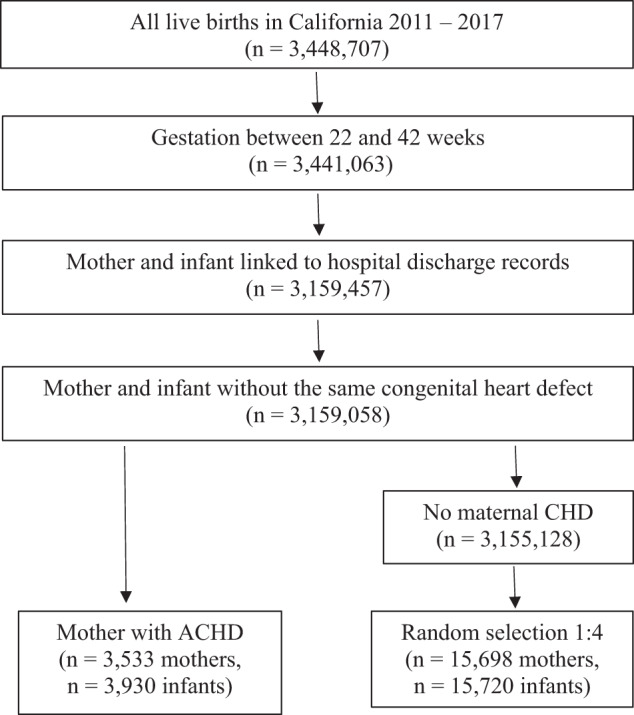
Sample Selection.

We utilized a database containing all live births in California from 2011–2017. Birth and infant death certificates, maintained by California Vital Statistics, were linked to a hospital discharge, emergency department, and ambulatory surgery records maintained by the California Department of Health Care Access and Information (HCAI). All licensed hospitals in California report discharge information to HCAI. This database contains information from records for maternal visits one year before birth and maternal and infant hospitalizations (including birth) and readmissions from birth to 1 year of age. Linkage was done using probabilistic matching. The database includes 25 diagnosis and 21 procedure codes per admission based on the *International Classification of Diseases, Ninth Revision, Clinical Modification* (ICD-9-CM) and the *International Classification of Diseases, Tenth Revision, Clinical Modification* (ICD-10-CM). We have used this database in several studies examining pregnancy and neonatal outcomes [30–32]. We included all infants with gestational ages (GA) from 22 to 44 weeks and their respective mothers (*n* = 3,441,063). (Fig. 1).

### Subjects

We identified all infants born to a mother with ACHD and randomly selected 4 mothers without ACHD for each mother with ACHD from the rest of the cohort. Mothers with ACHD were identified based on ICD-9-CM or ICD-10-CM codes present in maternal hospital records (Supplemental Table 1). To avoid including mothers who had their infants’ intrauterine diagnosis of CHD in their chart rather than their own disease, we excluded cases in which an identical code for CHD was used in the maternal and infant chart without another code in the maternal chart indicating a personal history of congenital heart disease (*n* = 399). (Fig. 1).

### Outcomes

To investigate the first aim of the study, the following pregnancy outcomes were assessed: prematurity (GA less than 37 weeks) and critical congenital heart disease (CCHD) of the infant. CCHD of the infant was identified using ICD-9 and ICD-10 codes, defining CCHD as congenital heart disease that is likely to be detected on pulse oximetry screening in the neonatal period most of the time (Supplemental Table 2).

To investigate the second aim of the study, the following infant outcomes were assessed: 1-year mortality, major neonatal morbidities, need for mechanical ventilation after birth, persistent pulmonary hypertension of the newborn (PPHN), prolonged birth hospitalization, and rehospitalization during the first year of life. Mortality was ascertained through death certificates or hospital discharge status. Major neonatal morbidities included bronchopulmonary dysplasia (BPD, ICD-9-CM 770.7, ICD-10-CM P27.1), necrotizing enterocolitis (NEC, ICD-9-CM 777.5, ICD-10-CM P77), intraventricular hemorrhage>grade II (IVH, ICD-9-CM 772.13 and 772.14, ICD-10-CM P52.2), periventricular leukomalacia (PVL, ICD-9-CM 779.7, ICD-10-CM P91.2), and retinopathy of prematurity (ROP) > stage 2 (ICD-9-CM 362.25-7 or 14.2, 14.5, 14.7 14.9, ICD-10-CM H35.14-6 or any procedure code for surgery on the retina or choroid plexus). Mechanical ventilation was defined as any type of positive-pressure ventilation (ICD-9-CM 93.90, 93.91, 96.7, 96.04, V46.1, ICD-10-CM 5A10, 5A093, 5A094, 5A095, 0BH1). PPHN was defined with the following ICD-9-CM and ICD-10-CM codes: 416.0, 416.8; P29.3, I27.0, I27.2. Prolonged birth hospitalization was defined as length of stay longer than two days for infants delivered via vaginal delivery, or more than four days for those delivered via c-section. Hospital readmissions were found by linking infant discharge records throughout the first year of life.

### Covariates

For the analysis of pregnancy outcomes, MMS, MPS [20–22], pregnancy infection, and maternal CHF were identified a priori as potential mediators based on the existing literature [20–28]. MMS was defined as the presence of one or more of the following conditions: preexisting maternal hypertension (ICD-9-CM 642.0, 642.1, 642.2, ICD-10-CM O10.x), dysmetabolic syndrome (ICD-9-CM: 277.7, ICD-10-CM: E 88.81), dyslipidemia (ICD-9-CM: 272.0, 272.1, 272.2, 727.4, 727.5, ICD-10-CM: E78.0, E78.1, E78.2, E78.4, E78.5), or type 2 diabetes mellitus (ICD-9-CM 250 specified as type 2, ICD-10-CM E11). MPS was defined as the presence of preeclampsia (ICD-9-CM 642.4, 642.5, 642.6, 642.7 ICD-10-CM: O11, O14.0, O14.9, O14.1, O14.2, O15), gestational hypertension (ICD-9-CM 642.3, ICD-10-CM O13), placental infarction (ICD-10-CM O43.81), or placental abruption (ICD-9-CM 641.2, 762.1 ICD-10-CM 045.0, 045.8, 045.9, P0.21). Pregnancy infection was defined as any infection complicating pregnancy (ICD-9-CM 646.5, 646.6, 647, ICD-10-C O23, O98), and chorioamnionitis (ICD-9-CM 762.7, ICD-10-CM O41.12). Maternal CHF was defined by the following ICD codes: ICD-9-CM 428, ICD-10-CM I50.

For the analysis of the association of ACHD and neonatal outcomes, we identified prematurity, CCHD and major birth defects (MBD) or chromosomal abnormalities as potential mediators [33]. Structural birth defects were considered major if they were determined by clinical review to result in mortality or major morbidity and likely to be identified at birth or lead to hospitalization during the first year of life. Chromosomal abnormalities were defined by ICD codes (ICD-9-CM 759, ICD-10-CM Q9).

### Statistical analysis

A Chi-squared test was used to compare baseline characteristics between maternal ACHD and no maternal ACHD. For pregnancy outcomes as well as for neonatal outcomes, univariable and multivariable logistic regression analyses were performed for each outcome and 95% confidence intervals (CI) were calculated. For pregnancy outcomes (i.e. prematurity and offspring with CCHD), we adjusted for MPS, metabolic syndrome, pregnancy infection and maternal CHF. For neonatal outcomes (i.e. 1-year mortality, major neonatal morbidity, need for mechanical ventilation, long birth hospitalization, and rehospitalization during the first year of life), we adjusted for GA, sex, small for gestational age (defined as birth weight <10th percentile for GA and sex using the method proposed by Talge et al., Pediatrics 2014) [33], CCHD, other malformations or syndromes and multiple gestation.

Mediation analyses were then performed using structural equation modeling. Mediators are defined as a variable on a proposed causal pathway between a predictor and outcome. To perform a mediation analysis, three conditions must be met: (1) the predictor must be significantly associated with the outcome, (2) the predictor must be significantly associated with the mediator and (3) the mediator must be significantly associated with the outcome. These conditions were confirmed by logistic regression (association between predictor and outcomes, as described above), chi-squared analysis (association between predictor and potential mediators) and multivariate logistic regression (association between potential mediators and outcomes). A more detailed description of mediation analyses is included in the supplemental section. For aim 1, our primary predictor was maternal ACHD and potential mediators were MPS, MMS, pregnancy infection and maternal CHF, and our outcomes were prematurity and neonatal CCHD. For aim 2, our primary predictor was maternal ACHD, potential mediators were prematurity, the presence of birth defects or chromosomal anomalies, and neonatal CCHD. Our outcomes were 1-year mortality, major neonatal morbidity, need for mechanical ventilation, long birth hospitalization, or readmission during the first year of life. If the necessary conditions were met for that variable, structural equation modeling was used to calculate the proportion of the total effect mediated. Confidence intervals were calculated using bootstrapping.

All analyses were performed by using the Mediation package in Stata version 16.2 (Stata Statistical Software: Release 16. College Station, TX: StataCorp LP). Code available upon request.

## Results

We identified 3930 neonates born to 3533 mothers with ACHD and matched them to 15,720 neonates born to 15,698 mothers without ACHD from the rest of the cohort. (Fig. 1).

### Analyses for pregnancy outcomes

Mothers with ACHD had an increased risk of preterm birth (13.9% versus 8.4%, *p* < 0.001) and of having an offspring with CCHD (1.8% versus 0.4%, *p* < 0.001) meeting the first condition for the mediation analysis (Table 1).

**Table 1:** Maternal and neonatal characteristics.

Maternal characteristics		No maternal CHD	Maternal CHD	*p*-value
*N*		15 698	3 533	
Maternal age	<18 years	264 (1.7%)	56 (1.6%)	0.90
	18–34 years	12 222 (77.9%)	2 759 (78.1%)	
	>34 years	3 212 (20.5%)	718 (20.3%)	
Parity	Nulliparous	5 911 (37.7%)	1 635 (46.3%)	<0.001
	Multiparous	9 787 (62.3%)	1 898 (53.7%)	
Delivery Mode	Vaginal delivery	10 294 (65.6%)	2 079 (58.8%)	<0.001
	C-section	5 404 (34.4%)	1 454 (41.2%)	
MPS^a^	No	14 434 (91.9%)	3 047 (86.2%)	<0.001
	Yes	1 264 (8.1%)	486 (13.8%)	
Preeclampsia	No	15 015 (95.6%)	3 247 (91.9%)	<0.001
	Yes	683 (4.4%)	286 (8.1%)	
Gestational hypertension	No	15 244 (97.1%)	3 389 (95.9%)	<0.001
	Yes	454 (2.9%)	144 (4.1%)	
Placental abruption	No	15 555 (99.1%)	3 471 (98.2%)	<0.001
	Yes	143 (0.9%)	62 (1.8%)	
Metabolic syndrome^b^	No	15 339 (97.7%)	3 316 (93.9%)	<0.001
	Yes	359 (2.3%)	217 (6.1%)	
Type 2 diabetes	No	15 616 (99.5%)	3 490 (98.8%)	<0.001
	Yes	82 (0.5%)	43 (1.2%)	
Preexisting hypertension	No	15 472 (98.6%)	3 392 (96.0%)	<0.001
	Yes	226 (1.4%)	141 (4.0%)	
Dyslipidemia	No	15 633 (99.6%)	3 485 (98.6%)	<0.001
	Yes	65 (0.4%)	48 (1.4%)	
Maternal Obesity	No	12 352 (78.7%)	2 774 (78.5%)	0.83
	Yes	3 346 (21.3%)	759 (21.5%)	
Congestive heart failure	No	15 682 (99.9%)	3 409 (96.5%)	<0.001
	Yes	16 (0.1%)	124 (3.5%)	
Infection during pregnancy	No	13 617 (86.7%)	2 784 (78.8%)	<0.001
	Yes	2 081 (13.3%)	749 (21.2%)	
Chorioamnionitis	No	15 227 (97.0%)	3 369 (95.4%)	<0.001
	Yes	471 (3.0%)	164 (4.6%)	
Other infection	No	13 997 (89.2%)	2 896 (82.0%)	<0.001
	Yes	1 701 (10.8%)	637 (18.0%)	
*Neonatal characteristics*				
*N*		15 720	3 930	
Sex	female	7 651 (48.7%)	1 934 (49.2%)	0.54
	male	8 069 (51.3%)	1 996 (50.8%)	
Prematurity	No	14 406 (91.6%)	3 382 (86.1%)	<0.001
	Yes	1 314 (8.4%)	548 (13.9%)	
Multiple Gestation	No	15 242 (97.0%)	3 786 (96.3%)	0.046
	Yes	478 (3.0%)	144 (3.7%)	
SGA	No	14 299 (91.0%)	3 425 (87.2%)	<0.001
	Yes	1 421 (9.0%)	505 (12.8%)	
CCHD	No	15 656 (99.6%)	3 858 (98.2%)	<0.001
	Yes	64 (0.4%)	72 (1.8%)	
Other major malformations or chromosomal anomalies	No	15 401 (98.0%)	3 794 (96.5%)	<0.001
	Yes	319 (2.0%)	136 (3.5%)	

^a^Maternal Placental Syndrome, consisting of any of the following: preeclampsia, gestational hypertension, placental abruption.

^b^Metabolic Syndrome consisting of any of the following: type 2 diabetes, preexisting hypertension, dyslipidemia, maternal obesity.

Further, mothers with ACHD had increased incidence of MPS (13.8% vs. 8.1%, *p* < 0.001), MMS (6.1% vs. 2.3%, *p* < 0.001), pregnancy infections (21.2% vs. 13.3%, *p* < 0.001) and maternal CHF (3.5% vs 0.1%, *p* < 0.001) fulfilling the second condition (Table 1).

**Fig. 2:**
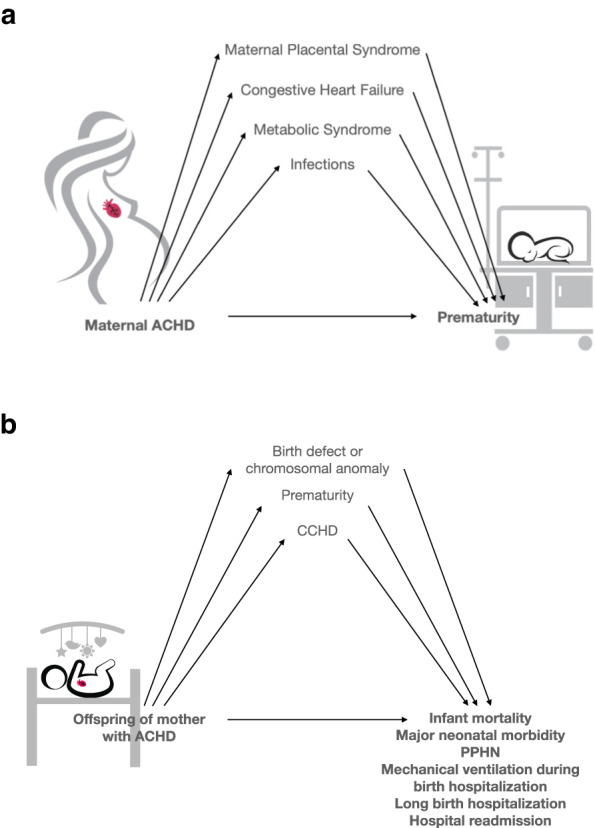
Conceptual model for the mediation analysis performed. **a** Mediation analysis for association of adult congenital heart disease and prematurity. **b** Mediation analysis for association of adult congenital heart disease and various neonatal outcomes. ACHD adult congenital heart disease, CCHD critical congenital heart disease, PPHN persistent pulmonary hypertension of the newborn.

Table 2 assesses condition three of the mediation analysis. It shows the results of the univariable and multivariable analysis assessing pregnancy outcomes. Mothers with MPS, MMS, pregnancy infection, and CHF were all at increased odds of giving birth prematurely. However, mothers with MPS, MMS, pregnancy infection, and maternal CHF were not at increased odds of giving birth to an infant with CCHD (Table 2). Thus, MPS, MMS, pregnancy infection and maternal CHF qualified as mediators in the relationship between ACHD and prematurity (Fig. 2a). Maternal CHF also qualified as a mediator in a relationship between ACHD and neonatal CHD; however, given that none of the other potential mediators fulfilled condition three and it felt to be unlikely that maternal CHF was on the causal pathway between ACHD and neonatal CHD, further mediation analysis using neonatal CHD as an infant outcome was not pursued.

**Table 2:** Univariable and multivariable analysis for pregnancy outcomes.

	Prematurity		Offspring with CCHD	
	Crude OR (95% CI)	Adjusted OR^a^ (95% CI)	Crude OR (95% CI)	Adjusted OR^a^ (95% CI)
Maternal ACHD	1.69 (1.51–1.89)	1.34 (1.19–1.51)	4.79 (3.40–6.76)	4.74 (3.34–6.74)
MPS	4.92 (4.37–5.54)	4.59 (4.07–5.18)	1.48 (0.89–2.47)	1.21 (0.72–2.04)
Metabolic Syndrome	2.12 (1.70–2.64)	1.76 (1.39–2.22)	1.82 (0.85–3.92)	1.28 (0.59–2.78)
Pregnancy infection	1.60 (1.42–1.81)	1.40 (1.23–1.59)	0.97 (0.60–1.59)	1.19 (0.37–3.84)
Maternal CHF	6.31 (4.47–8.89)	3.60 (2.47–5.23)	3.22 (1.01–10.24)	0.78 (0.48–1.28)

*CHD* congenital heart disease, *MPS* maternal placental syndrome, *CHF* congestive heart failure, *OR* odds ratio, *CI* confidence interval.

^a^adjusted for all other mediators listed in the table.

Mediation analysis demonstrated that 21.9% (95% CI 16.6–30.1%) of the total effect of maternal ACHD on prematurity was mediated through MPS, 14.3% by maternal CHF (95% CI 9.0–21.3%), 5% by pregnancy infection (95% CI 3.0–8.2%), and 4.4% by MMS (95% CI 2.3–7.5%). A direct effect from maternal ACHD accounted for 54.4% of the total relationship between maternal ACHD and prematurity (Table 3).

**Table 3:** Mediation analysis for maternal CHD and prematurity.

	Proportion of total effect mediated (95% CI^a^)
MPS	21.9 (16.6–30.1)
Metabolic Syndrome	4.4 (2.3–7.5)
Pregnancy infection	5.0 (3.0–8.2)
Maternal CHF	14.3 (9.0–21.3)
Direct effect from mat CHD	54.4 (40.0–64.3)

^a^bias corrected.

### Analyses for neonatal outcomes

Infants of mothers with ACHD had an increased crude odds of death in the first year of life (OR 2.32, 95% CI 1.47–3.69), though this effect became insignificant after adjustment (adjusted OR 1.27, 95% CI 0.77–2.07). Infants of mothers with ACHD had increased odds of major neonatal morbidity, need for mechanical ventilation, prolonged birth hospitalization, and rehospitalization during first year of life (Table 4). Thus, all these associations meet condition 1 of the mediation analysis.

**Table 4:** Univariable and multivariable analysis for neonatal outcomes.

	No maternal CHD (*n* = 15,720)	Maternal CHD (*n* = 3930)	Crude OR (95% CI)	Adjusted^c^ OR (95% CI)
1-year mortality	0.3%	0.7%	2.32 (1.47–3.69)	1.27 (0.77–2.07)
Major neonatal morbidity^a^	0.7%	1.7%	2.48 (1.82–2.28)	1.53 (1.10–2.14)
PPHN	0.3%	0.8%	2.57 (1.62–4.06)	1.57 (0.96–2.60)
Need for NIPPV or IPPV during birth hospitalization	3.7%	7.9%	2.21 (1.91–2.55)	1.64 (1.40–1.92)
Long birth hospitalization^b^	12.4%	20.1%	1.77 (1.62–1.94)	1.48 (1.34–1.64)
Hospital readmission during first year of life	10.7%	13.5%	1.30 (1.17–1.49)	1.13 (1.01–1.26)

*PPHN* persistent pulmonary hypertension of the newborn, *NIPPV* non-invasive positive pressure ventilation, *IPPV* invasive positive pressure ventilation, *OR* odds ratio, *CHD* congenital heart disease, *CI* confidence interval.

^a^Defined as one of the following: bronchopulmonary dysplasia, severe retinopathy of prematurity requiring intervention, intraventricular hemorrhage >grade 2, necrotizing enterocolitis.

^b^For vaginal delivery length of stay >2 days, for c-section length of stay >4 days.

^c^adjusted for gestational age, sex, small for gestational age, critical congenital heart disease, and other malformations or syndromes.

Infants of mothers with ACHD had an increased risk of prematurity (13.9% vs. 8.4%, *p* < 0.001), CCHD (1.8% vs 0.4%, *p* < 0.001), and having a MBD (3.5% vs. 2.0%, *p* < 0.001) (Table 1) fulfilling condition 2 for these mediators.

Each of the potential mediators was significantly associated with each of the neonatal outcomes (Supplemental Table 3, condition 3).

Mediation analysis for each neonatal outcome was then performed with all qualifying mediators (Fig. 2b). This demonstrated that 28.6% (95% CI 16.3–63.6%) of the effect of maternal ACHD on 1-year mortality was mediated by prematurity, 28.1% (95% CI 12.4–70.2%) by CCHD in the infant, with a lesser effect from MBD (9.9%, 95% CI 3.6–25.8%), and some direct effect of maternal ACHD (33.3%, 95% CI −33.7–61.3%) (Table 5). The effect of ACHD on major neonatal morbidities was mediated by prematurity (40.1%, 95% CI 28.3–63.7), with a large direct effect from maternal ACHD (47.1%, 95% CI 20.5–62.9%). Mediation analyses of ACHD and the need for mechanical ventilation, and long birth hospitalization demonstrated some effect from prematurity and CCHD, but also a large direct effect from maternal ACHD. A significant portion of the effect of ACHD on PPHN and hospital readmission during the first year of life was mediated by CCHD in the infant. (Table 5).

**Table 5:** Mediation analysis for neonatal outcomes.

	Prematurity^a^	CCHD in offspring^a^	MBD or chromosomal anomaly^a^	Direct effect of maternal CHD^a^
1-year mortality	28.6 (16.3–63.6)	28.1 (12.4–70.2)	9.9 (3.6–25.8)	33.3 (−33.7–61.3)
Major neonatal morbidity^b^	40.1 (28.3–63.7)	4.2 (0.0–10.9)	8.6 (4.4–16.2)	47.1 (20.5–62.9)
PPHN	10.2 (4.6–29.5)	37.1 (17.6–87.7)	6.9 (2.4–25.1)	45.8 (−28.7–69.5)
Need for NIPPV or IPPV during birth hospitalization	28.6 (21.7–36.7)	13.3 (9.1–19.6)	4.4 (2.6–7.5)	53.7 (42.2–62.1)
Long birth hospitalization^c^	30.6 (24.3–37.6)	6.6 (4.5–10.1)	3.4 (1.7–5.3)	59.4 (52.1–67.3)
Hospital readmission during first year of life	10.1 (5.8–19.3)	27.7 (16.9–45.8)	19.8 (11.1–36.5)	42.3 (2.5–60.1)

*CCHD* critical congenital heart disease, *MBD* multiple birth defects, *CHD* congenital heart disease, *PPHN* persistent pulmonary hypertension of the newborn, *NIPPV* noninvasive positive pressure ventilation, *IPPV* invasive positive pressure ventilation.

^a^Proportion of total effect mediated (95% CI).

^b^Defined as one of the following: bronchopulmonary dysplasia, severe retinopathy of prematurity requiring intervention, intraventricular hemorrhage > grade 2, necrotizing enterocolitis.

^c^For vaginal delivery length of stay >2 days, for c-section length of stay >4 days.

## Discussion

In this population-based case-control study, we showed an increased risk of adverse outcomes in offspring born to mothers with ACHD including prematurity, 1-year mortality, major neonatal morbidity, need for mechanical ventilation, long birth hospitalization and hospital readmission during the first year of life. In mediation analyses, we identified MPS and maternal CHF as important mediators in the relationship between maternal ACHD and prematurity. Prematurity, CCHD and MBD mediated the association between maternal ACHD and infant outcomes. However, the direct effect of ACHD on pregnancy and infant outcomes not explained by the mediators assessed in this study was significant.

Pregnancy outcomes in this report are in line with previous studies. Drenthen et al. reported rates of prematurity of 15.9% and CCHD in the offspring of 3.5%, compared to 13.9% and 1.8%, respectively, in our population [12]. In the mediation analysis between ACHD and prematurity, MPS was the most important mediator. In 2005, Ray et al. defined MPS as hypertensive disorders of pregnancy and placental abruption or infarction, showing that women with MPS during pregnancy had higher cardiovascular morbidity [20]. There is an epidemiological link between MPS and CHD: Auger et al. showed that women with preeclampsia are more likely to carry a fetus with CHD [34]. Interestingly, women with ACHD also are more likely to develop preeclampsia during pregnancy [14]. It is possible that women with ACHD have vascular imbalances driven by chronic hypoxia that led to a higher risk of MPS. The second most important mediator of prematurity was maternal CHF, explaining 14.3% of the effect between ACHD and prematurity. Severe CHF might be an indication to induce labor before a fetus reaches full-term, or this may be a result of insufficient placental oxygen delivery [35]. The direct effect of ACHD on prematurity remained large in our study at 54.4%. Thus, there are likely other mediators that we did not assess that contribute to increased rates of prematurity in mothers with ACHD. Identifying these factors should be subject of further study.

We report similar rates of mortality to previous studies. Ramage et al. to be 0.8% mortality, compared to 0.7% in our study [11]. Given that our database included information beyond the neonatal period, we were able to investigate some important outcomes during the first year of life of infants born to mothers with ACHD. While the increased risk of 1-year mortality and PPHN were statistically insignificant after adjusting for covariates including prematurity, the increased risk of major neonatal morbidity, need for respiratory support, long birth hospitalization and hospital readmission during the first year of life remained significant.

In mediation analysis, prematurity, CCHD in the offspring and MBD largely explained the increased 1-year mortality and the increased rate of PPHN. Prematurity, CCHD and MBD/chromosomal anomalies have all been shown to have strong associations with mortality and as such, this finding is expected [30, 36, 37]. However, surprisingly, while our mediators all explained a portion of the effect of ACHD on the rest of the neonatal outcomes, the direct effect of ACHD was significant. Prematurity, CCHD in the offspring, and MBD/chromosomal anomalies together only explained approximately 50% of the increased risk for neonatal morbidities, need for respiratory support, longer birth hospitalization and hospital readmissions during the first year of life. A direct effect of maternal ACHD on infant outcomes explained the rest. A large direct effect may suggest that there are likely other mediators not assessed in this study that explain these results. Factors that were not evaluated as mediators that have been describe as affecting placental and neonatal health in congenital heart disease include subclinical placental microthromboses, fetal vascular malperfusion from abnormal flow, cardiac insufficiency, and hyperviscosity due to chronic hypoxia and resultant avascular villi in the placenta, medication effects, and socioeconomic factors that could potentially limit access to care for both the mother and the infant [38, 39]. These potential mediators that were unable to be assessed in this study warrant further investigation. Notably, 95% confidence interval for the direct effect of maternal CHD on 1-year mortality and PPHN include 0%. While this may suggest that the direct effect of maternal CHD may be negligible, it also is a reflection of the relatively low rate of mortality and PPHN and the resultant wide confidence intervals of these effects in our analysis. It is crucial to realize though, that prematurity, CCHD and MBD are not the primary drivers of morbidity in these infants in the first year of life. Further studies should aim at identifying factors that explain the high vulnerability of infants born to mothers with ACHD.

The strengths of this study stem from the use of a large population-based dataset that allows for linkage of maternal conditions to neonatal and infant outcomes up to 1 year of age. However, many of the limitations are also inherent to the use of a database such as this one. The identification of mothers with ACHD relied upon accurate documentation of ICD codes. However, only 35–44% of patients with ACHD can describe their heart condition [40–42], and in one statewide database sample of hospitalizations involving patients with ACHD, 75% of patients with simple ACHD had no CHD diagnosis recorded in the hospitalization [43]. Thus, we must assume that there is underreporting of maternal ACHD, particularly mild ACHD, in this database and that our sample of mothers with ACHD is likely skewed towards more severe cardiac disease. We also had limited treatment data available; therefore, it is unclear to what degree increased rates of prematurity were due to early induction of labor. Furthermore, certain medications commonly prescribed to patients with ACHD (i.e. beta blockers) are known to be associated with preterm delivery [6], and the degree to which prematurity was mediated by these factors cannot be determined. A further limitation of our study is that only live-born pregnancies were included, as the database used in this study does not contain information about pregnancies that resulted in fetal demise or termination of pregnancy, and therefore we may be underestimating the extent to which maternal ACHD influences fetal health. Nonetheless, we believe that the results of our mediation analysis contribute to understanding the complex interplay between ACHD, pregnancy and infant outcomes.

## Conclusion

We showed that neonates born to mothers with ACHD are at higher risk for adverse outcomes including 1-year mortality, major neonatal morbidity, need for mechanical ventilation, long birth hospitalization and hospital readmissions during the first year of life. The mediation analyses identified MPS and maternal CHF as significant factors explaining the effect between ACHD and prematurity. Prematurity itself, together with CCHD and MBD in the offspring, explain the increased 1-year mortality in infants born to mothers with ACHD, however, the direct effect of ACHD on other outcomes remained significant, raising the question of what other factors are driving these outcomes.

### Supplementary information

Supplemental Information, Supplemental Table 1, Supplemental Table 2, Supplemental Table 3

## Data Availability

The data use agreement with the California Department of Health Care Access and Information prohibits distribution of any patient-level data; thus, the data used for this study are not made publicly available. Data can be requested from Department of Health Care Access and Information (https://hcai.ca.gov/) by qualified researchers for a fee.
