# Supplementary material for: What drives outcomes in infants of mothers with congenital heart disease? A mediation analysis
Source: J Perinatol. 2023 Oct 19;44(3):366–72. doi: 10.1038/s41372-023-01796-0 (PMC10920192; doi:10.1038/s41372-023-01796-0)
Supplement: Supplementary file 1 — Supplemental Information, Supplemental Table 1, Supplemental Table 2, Supplemental Table 3 [file 41372_2023_1796_MOESM1_ESM.docx]

**Supplemental Information: Mediation analysis**

A mediation analysis seeks to explain the relationship between a predictor and an outcome via the inclusion of a third “mediator” variable. A visual model of this relationship is depicted here. The mediator is defined as a variable on causal pathway between the predictor and outcome. Mediation analyses seek to further elucidate the mechanism of the relationship between a predictor and an outcome when their relationship is not immediately clear. In order to perform a medication analysis, three conditions must be established. First, the predictor must be significantly associated with the outcome. Establishing this relationship is important because it demonstrates that there is an effect that a third variable can mediate. Similarly, steps two and three establish that the predictor is significantly associated with the mediator and the mediator is significantly associated with the outcome. If these conditions are met, structural equation modeling can then be used to calculate the proportion of the total effect mediated by the mediator variable. Subsequently, confidence intervals can be calculated using bootstrapping.


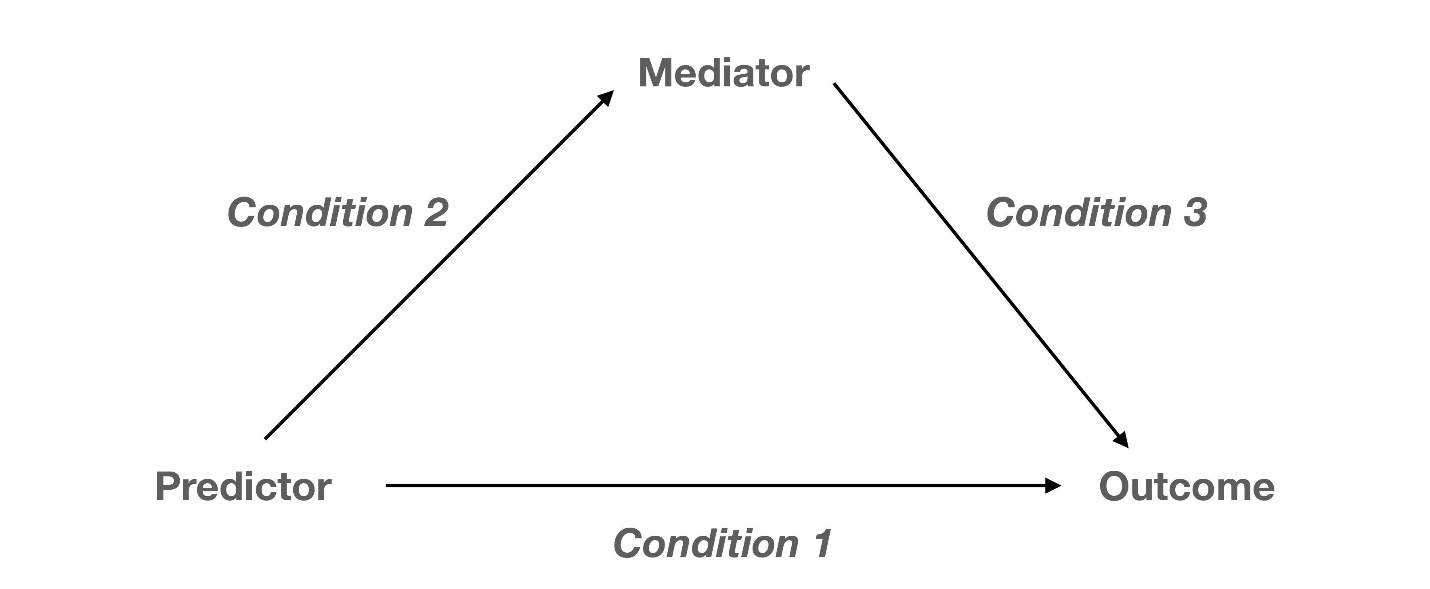


**Supplemental Table 1: ICD-9 and ICD-10 codes used to identify maternal congenital heart disease**

| **ICD-9** |  |
| --- | --- |
| 745 | Bulbus cordis anomalies and anomalies of cardiac septal closure |
| 746 | Other congenital anomalies of the heart |
| 747.1 | Coarctation of the aorta |
| 747.2 | Other congenital anomalies of the aorta |
| 747.3 | Congenital anomalies of pulmonary artery |
| 747.4 | Congenital anomalies of great veins |
| 648.5 | Congenital cardiovascular disorders complicating pregnancy childbirth or the puerperium |
| V13.65 | Personal history of (corrected) congenital malformations of heart and circulatory system. |
| **ICD-10** |  |
| Q20 | Congenital malformations of cardiac chambers and connections |
| Q21 | Congenital malformations of cardiac septa |
| Q22 | Congenital malformations of pulmonary and tricuspid valves |
| Q23 | Congenital malformations of aortic and mitral valves |
| Q24 | Other congenital malformations of heart |
| Q25.1 | Coarctation of aorta |
| Q25.2 | Atresia of aorta |
| Q25.3 | Supravalvular aortic stenosis |
| Q25.4 | Other congenital malformations of aorta |
| Q25.5 | Atresia of pulmonary artery |
| Q25.6 | Stenosis of pulmonary artery |
| Q25.7 | Other congenital malformations of pulmonary artery |
| Q25.8 | Other congenital malformations of other great arteries |
| Q25.9 | Congenital malformation of great arteries, unspecified |
| Q26 | Congenital malformations of the great veins |
| Z87.74 | Personal history of (corrected) congenital malformations of heart and circulatory system |

**Supplemental Table 2: ICD-9 and ICD-10 codes used to identify infant critical congenital heart disease**

| **ICD-9** |  |
| --- | --- |
| 745.0 | Common truncus |
| 745.1 | Transposition of the great vessels |
| 745.2 | Tetralogy of Fallot |
| 745.3 | Common ventricle |
| 745.6 | Endocardial cushion defect |
| 746.0 | Anomalies of the pulmonary valve |
| 746.1 | Tricuspid atresia and stenosis, congenital |
| 746.2 | Ebstein’s anomaly |
| 746.3 | Aortic valve stenosis |
| 746.7 | Hypoplastic left heart syndrome |
| 747.1 | Coarctation of aorta |
| 747.4 | Congenital anomalies of great veins |
| **ICD-10** |  |
| Q20.0 | Common arterial trunk |
| Q20.3 | Double outlet right ventricle |
| Q20.4 | Double inlet ventricle |
| Q21.2 | Atrioventricular septal defect |
| Q21.3 | Tetralogy of Fallot |
| Q22.0 | Pulmonary valve atresia |
| Q22.1 | Congenital pulmonary valve stenosis |
| Q22.2 | Congenital pulmonary valve insufficiency |
| Q22.3 | Other congenital malformations of pulmonary valve |
| Q22.4 | Congenital tricuspid stenosis |
| Q22.5 | Ebstein’s anomaly |
| Q22.6 | Hypoplastic right heart syndrome |
| Q22.8 | Other congenital malformations of tricuspid valve |
| Q22.9 | Congenital malformation of tricuspid valve, unspecified |
| Q23.0 | Congenital stenosis of aortic valve |
| Q23.4 | Hypoplastic left heart syndrome |
| Q25.1 | Coarctation of the aorta |
| Q25.3 | Supravalvular aortic stenosis |
| Q26 | Congenital malformations of great veins |

**Supplemental Table 3:** Univariable analysis for neonatal outcomes^1^

|  | Infant Mortality | Major Neonatal Morbidity | PPHN | Mechanical ventilation during birth hospitalization | Long birth hospitaliz-ation | Hospital readmission in first year of life |
| --- | --- | --- | --- | --- | --- | --- |
| Birth defect or chromosomal abnormality | 11.1 (6.3, 19.3) | 11.7 (8.0, 17.1) | 8.0 (4.3, 14.9) | 5.7 (4.5, 7.3) | 4.1 (3.3, 4.9) | 8.6 (7.1, 10.4) |
| Prematurity | 14.4 (9.1, 22.6) | 46.6 (31.5, 68.9) | 4.6 (2.9, 7.5) | 13.1 (11.4, 15.1) | 11.5 (10.4, 12.8) | 1.8 (1.6, 2.1) |
| Congenital heart disease | 28.1 (14.8, 53,2) | 5.4 (2.4, 12.4) | 46.3 (26.3, 81.7) | 18.7 (13.3, 26.5) | 8.5 (5.9, 12.1) | 15.6 (10.9, 22.2) |

^1^Crude odds ratios (95% CI)
